# IFN-α induced systemic lupus erythematosus complicated with hemophagocytic lymphohistiocytosis: a case report and literature review

**DOI:** 10.3389/fimmu.2023.1223062

**Published:** 2023-08-04

**Authors:** Zhipeng Zeng, Wei Tu, Bai Ji, Jie Liu, Kecheng Huang, Daan Nie, Liu Yang

**Affiliations:** ^1^ Department of Rheumatology and Immunology, Tongji Hospital, Tongji Medical College, Huazhong University of Science and Technology, Wuhan, China; ^2^ Department of Internal Medicine, the People Hospital of Tongshan, Xianning, China; ^3^ Department of Cardiology, Union Hospital, Tongji Medical College, Huazhong University of Science and Technology, Wuhan, China; ^4^ Department of Obstetrics and Gynecology, Tongji Hospital, Tongji Medical College, Huazhong University of Science and Technology, Wuhan, China; ^5^ Department of Cardiology, the Affiliated Hospital of Guizhou Medical University, Guiyang, China; ^6^ Department of Reproductive Medicine, Tongji Hospital, Tongji Medical College, Huazhong University of Science and Technology, Wuhan, China

**Keywords:** interferon-α, Peg-INFα-2b, systemic lupus erythematosus (SLE), Hemophagocytic lymphohistiocytosis (HLH), hepatitis B virus (HBV)

## Abstract

Hemophagocytic lymphohistiocytosis (HLH) is a severe and life-threatening hyperinflammatory condition characterized by excessive activation of macrophages and T cells and resulted in multi-organ dysfunction. HLH can be a primary disease or secondary to infections, malignancy, and some autoimmune diseases, including adult-onset Still’s disease (AOSD) and systemic lupus erythematosus (SLE). However, it is rare for HLH to occur as a secondary condition to drug-induced lupus erythematosus (DILE). In this report, we present a case of HLH as an unusual complication during SLE treatment in a 31-year-old male patient. The patient initially suffered from active chronic hepatitis B (CHB) and was treated with pegylated INFα-2b (Peg-INFα-2b), tenofovir disoproxil and lamivudine. After 19 months, CHB obtained biochemical and virological response with HBsAg positive to HBsAb. The patient developed fever, headache, and cytopenia after Peg-INFα-2b treatment for 33 months, and laboratory studies revealed that ANA and anti dsDNA were positive. He displayed 5 features meeting the HLH-2004 criteria for diagnosis including fever, pancytopenia, hyperferritinemia, high levels of soluble CD25, and hemophagocytosis on bone marrow biopsy. The patient was initiated with a combination treatment of intravenous methylprednisolone pulse therapy, oral cyclosporine, and etoposide (VP-16), which was followed by a course of oral prednisolone, intravenous cyclophosphamide pulse therapy, and entecavir with complete response. To our knowledge, this is the first report of IFN-α induced SLE complicating with HLH. Physicians should consider the potential autoimmune side effects of IFN-α therapy and be alert to insidious HLH in patients diagnosed with SLE.

## Introduction

Interferon-alpha (IFN-α) is a type of cytokines that is naturally produced by the immune system in response to viral infections, tumors, and other pathogens (1). When administered exogenously, IFN-α serves as a broad-spectrum antiviral and antitumor agent and is used in the treatment of hepatitis B and C, as well as other viral infections and some malignant neoplasm (2). IFN-α plays a crucial role in the body’s defense against viral infections to activate immune cells and inhibit viral replication. However, it is important to note that IFN-α injection can cause a range of side effects, including flu-like, weakness, nausea and vomiting, headaches, blood disorders, as well as liver damage or dysfunction. Most significantly, IFN-α may induce numerous unforeseen illnesses, such as thyroid disease, SLE, polymyositis, seronegative arthropathies, autoimmune thrombocytopenia, autoimmune hepatitis, and other conditions (1).

SLE is a prevalent autoimmune disease worldwide, affecting an estimated 0.40 million people annually with an incidence of 5.14 per 100,000 person-years (3). The occurrence of SLE varies among different populations, with an annual incidence of 8.82 (2.4 to 25.99) per 100,000 person-years in females and 1.53 (0.41 to 4.46) per 100,000 person-years in males, respectively (3). Although autoimmune induction is the primary cause of SLE, other contributing triggers have also been identified in the onset of the disease. Nonetheless, the use of medications, such as IFN-α therapy, is an increasingly significant factor that cannot be overlooked for the occurrence of SLE (4). IFN-α therapy rarely leads to SLE as an adverse reaction of drug use. The clinical manifestations of drug-induced lupus erythematosus (DILE) are similar to those of common lupus, including rash, lupus nephritis, fever, arthritis, and hematologic involvement (5). However, the complication of HLH has been rarely reported during the course of SLE progression.

Hemophagocytic syndrome, also referred to as HLH, is a fatal disease that is categorized into two types: primary and secondary HLH (6). Primary HLH (pHLH) is caused by the abnormal immune regulation of lymphocyte and proliferation and activation of macrophage, while secondary HLH (sHLH) can be triggered by underlying conditions such as viral, bacterial, or fungal infections, autoimmune diseases, or malignancies (6). The excessive activation of lymphocytes and macrophages result in the release of copious amounts of cytokines, leading to damage in multiple organs, including the liver, spleen, bone marrow, and central nervous system (6). HLH encompasses a broad range of symptoms, including fever, liver insufficiency, hepatosplenomegaly, cytopenia, and neurological symptoms such as seizures and confusion. The diagnose of HLH is always a challenge, as the symptoms can be similar to those of other conditions. The HLH-2004 criteria and HScore are recognized as the primary principles for HLH diagnosis (7, 8). A combination of clinical evaluation, laboratory tests, and imaging studies may be employed to make a diagnosis.

This study presents a case of a 31-year-old male patient with chronic hepatitis B (CHB) who was followed up for 2 years and developed SLE complicated with sHLH after receiving Peg-INFα -2b treatment. Here, we provide a detailed description of his clinical features and treatment history. In addition, we conducted a literature review to further promote the understanding of DILE and improve the efficacy of sHLH treatment.

## Case report

A 31-year-old male patient, who worked as a communication company employee, was admitted to our hospital on July 27, 2021, with a chief complaint of recurrent fever and headache for 3 months. The patient was diagnosed with CHB in October 2018, with positive results for HBsAg, HBeAg, and anti-HBC, and an HBV-DNA quantity of 1×10^7^ copies/ml, along with elevated transaminases (ALT 289U/L, AST 120U/L). Before IFN-α administration, the screening tests related to autoimmune disorder, including ANA and dsDNA, were conducted, and those were negative. Thus, he received Peg-IFNα-2b (180μg) subcutaneously every week and was also administrated with a combination of tenofovir disoproxil (300mg, QD) and lamivudine (100mg, QD). In September 2019, the patient’s HBsAg was negative, and the anti-HBs was positive, with ALT 34U/L and HBV-DNA<100 copies/ml, hence the therapeutic regime was maintained. Unexpectedly, in May 2021, the patient developed a headache, intermittent fever that reached 39.5°C, and reduced WBC (1230/μL). He was administered ibuprofen to reduce fever and received subcutaneous G-CSF (100 μg) treatment. However, despite the treatment, he continued to experience intermittent high-grade fever and his condition worsened over the five days prior to admission. In addition to the fever, he also had chills and 1-2 episodes of vomiting per day for three days. On July 10, the patient experienced severe persistent headache immediately after injection of 180μg Peg-IFNα-2b and was admitted to a local hospital. Laboratory tests revealed that WBC was 2330/μL, Leukocyte 670/μL, Hb 54g/L, PLT 27×10^4^/μL, ANA 1:1000, and anti-dsDNA positive (+). He was diagnosed with CHB and suspected of SLE, and treated with antipyretic, hepatoprotective, and anti-infective therapy, as well as intermittent transfusions of red blood cells and platelets. However, the patient’s fever persisted, and he was subsequently admitted to our hospital.

Physical examinations revealed that the patient had a body temperature of 36.3°C, a heart rate of 87 beats per minute, a respiratory rate of 19 beats per minute, and a blood pressure of 100/61 mmHg. The patient’s skin and mucous membranes were pale without any rash, bleeding spots, or oral ulcers. Laboratory tests showed that the patient’s hemoglobin level was 76g/L and platelet count was 67×10^4^/μL. The patient had a positive ANA (1:1000) with a nuclear homogeneous type, as well as positive anti-dsDNA antibody, anti-nucleosome antibody, and anti-histone antibody. Immunization detection showed that complement C_3_ was 0.52g/L (normal range: 0.65-1.39g/L), and the direct Coombs’ test was positive (++). The results of routine urine analysis, rheumatoid sets, antiphospholipid antibody, and ANCA were normal. Based on the patient’s clinical symptoms and laboratory findings, he met the 2019 ACR/EULAR Classification Criteria for SLE criteria and was diagnosed (9).The score of SLEDAI-2000 was 6, and suggested the active phase of disease.

**Figure 1:**
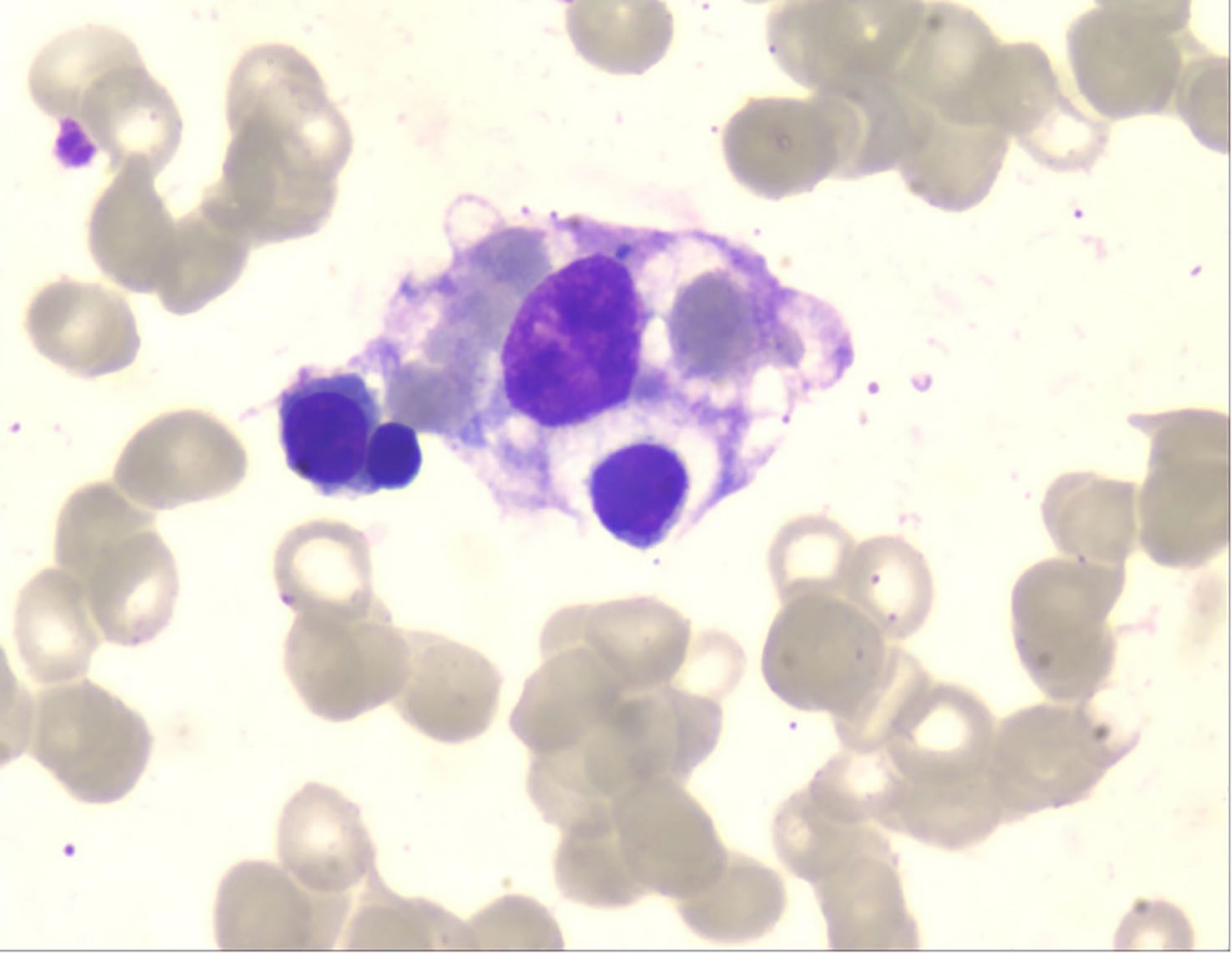
Bone marrow biopsy showing hemophagocytosis by histiocyte.

**Figure 2:**
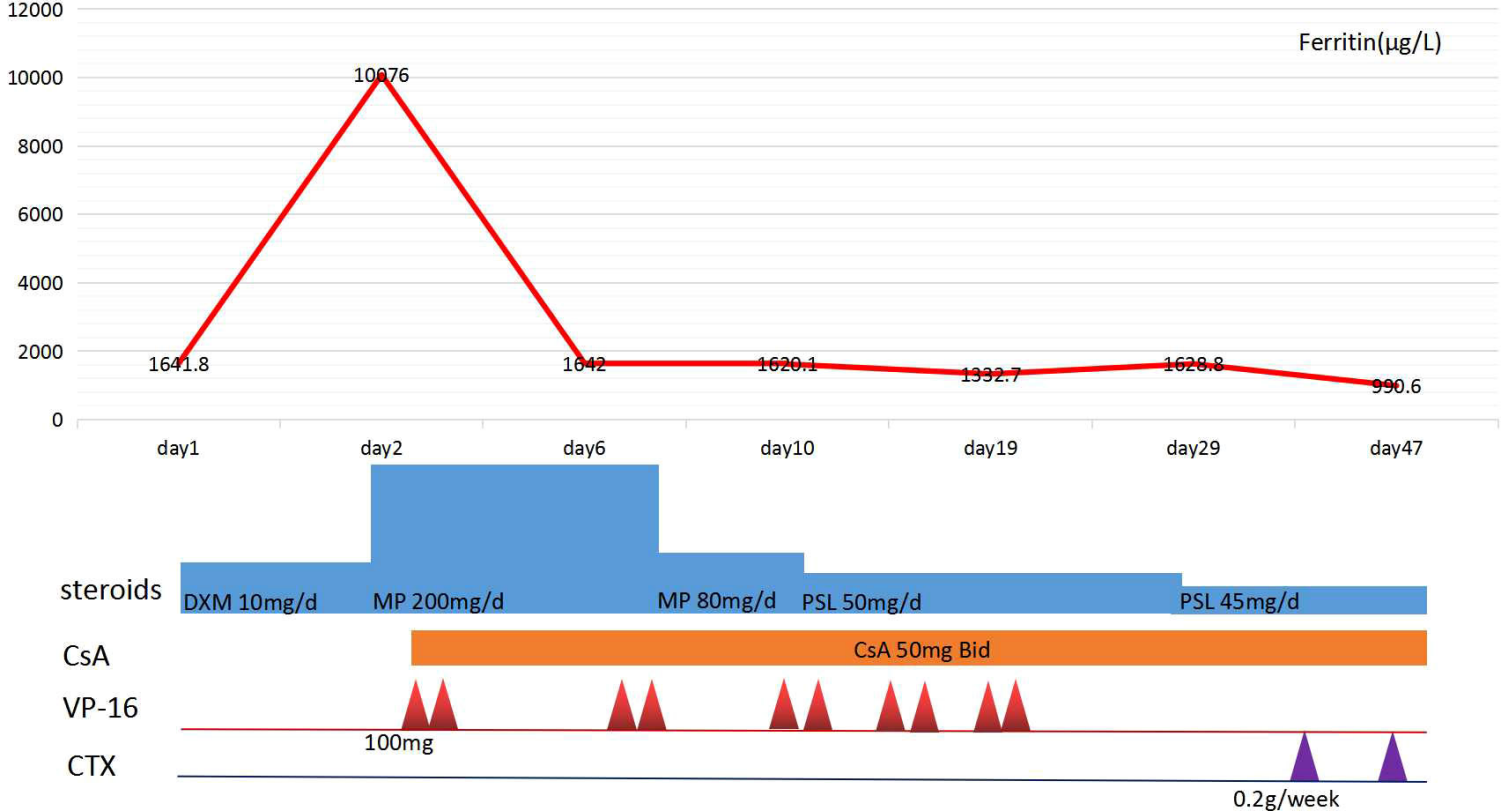
The patient’s clinical course in hospital. DXM, dexamethasone; MP, Methylprednisolone; PSL, prednisolone;VP-16,etoposide;CsA, Cyclosporine A;CTX, cyclophosphamide.

After the administration of 10 mg dexamethasone, the patient’s fever had persisted, with a highest temperature of 39.5°C. As shown in **Table 1**, laboratory results were significant for pancytopenia, hyperferritinemia, and elevated liver enzymes, with ALT 374U/L and AST 261U/L. His ferritin level was 10076 μg/L, and soluble CD25 (sIL-2R) was 8664.9 pg/ml (reference interval: 400-2500 pg/ml). Bone marrow biopsy revealed occasional hemophagocytic cells (**Figure 1**). The patient met the diagnosis criteria of HLH-2004, and the Hscore was 207, with a probability of 92% for HLH (8). Therefore, the patient was finally diagnosed with SLE complicated with secondary HLH, and 5 days of pulsed intravenous methylprednisolone with a dose of 200mg daily was administered for shock treatment. Subsequently, a combination treatment of etoposide (100mg×3/week, accumulated 1000mg), cyclosporine (50mg, bid), cyclophosphamide (200mg, qw), and entecavir was administered, which successfully reversed the patient’s body temperature to the normal range (**Figure 2**). After discharge, the patient received intravenous injections of cyclophosphamide (400mg) every 2 weeks, supplemented with reductive oral administrations of cyclosporine 50 mg twice daily and prednisone 50 mg once daily for outpatient therapy.The valley concentration of ciclosporin in patients was 208ng/ml after 2 weeks of administration.

**Table 1:** Laboratory data on fever after administration.

Parameters	Result	Reference interval
Complete blood counts		
White blood cells	4000/ul	3500-9500
Red blood cells	220*104/ul	430-580
Hemoglobin	7.1g/dl	13-17.5g/dl
Platelets	5.7*104/ul	12.5-35
Urinalysis		
Protein	–	–
Occult blood	–	–
Biochemistry		
hrCRP	28.85mg/L	0-5
Blood urea nitrogen	4.13mmol/L	3.1-8.0
Creatinine	53umol/L	59-104
Total protein	52.3g/L	64-83
Albumin	31.3g/L	35-52
Total bilirubin	10.0umol/L	≤16.8
glutamyltransferase	133U/L	10-71
Aspartate transaminase	374U/L	≤41
Alanine aminotransferase	261U/L	≤40
Lactate dehydrogenase	498U/L	135-225
Ferritin	10076ug/L	30-400
Triglyceride (mmol/l)	0.87mmol/l	≤1.7
Fibrinogen(g/l)	2.27g/l	2-4
Immunology		
Rheumatoid factor	6IU/ml	0-30
ANA	1:1000	–
Anti-dsDNA antibody	46.2IU/mL	0-10
C3	0.52g/L	0.8-1.8
C4	0.12g/L	0.1-0.4
IgG	12.79g/L	7-16
IL-6	2.69pg/ml	0.1-2.9
Soluble CD25	8664.9pg/ml	400-2500
Direct Coombs test	2+	–

Two months later, the patient was administered with prednisone (20mg, qd), and routine urine analysis, liver and kidney function, and ferritin were within normal ranges. The patient’s HBsAg and HBeAg were negative, and the anti-HBs was positive, with no detected HBV-DNA. Half year later, the ANA titer had decreased to 1:100, and the anti-nucleosome and anti-histone were negative, with normal complement levels. Given the stable condition, we recommend that the patient stop the therapy of cyclophosphamide. Until in February 2023, the patient remained stable and was receiving maintenance therapy consisting of prednisone 5mg, cyclosporine 50mg twice daily, entecavir 0.5 mg, and calcium tablets.

## Discussion

CHB, a liver disorder caused by long-term hepatitis B virus (HBV) infection, is characterized by a progressive deterioration of liver function, leading to cirrhosis, liver failure, and ultimately, liver cancer. The primary objective of CHB treatment is to inhibit HBV replication, reduce inflammation and necrosis in hepatocytes, and prevent the proliferation of liver fibrous tissue, so as to delay and minimize the occurrence of liver failure, cirrhosis decompensation, hepatocellular carcinoma (HCC), and other complications (10). The treatment modalities for HBV mainly consist of antiviral medications such as nucleoside analogs (NAs), IFN-α regimen intervention, as well as supportive therapy (11).

IFN-α has been approved for HBV treatment for several decades, with satisfactory remission rates (11). For instance, Peg-IFN-α has proven to be effective for HBV and may even reduce the incidence of HBV-associated HCC (12). However, it is worth noting that IFN-α administration may result in numerous side effects (2, 13). An unforeseen adverse response in HBV patients treated with IFN-α is the development of DILE. This underscores the significance of closely monitoring patients receiving IFN-α treatment for the possible onset of DILE and promptly discontinuing IFN-α if deemed necessary. To avoid side effect of IFN-α, it is very important to perform the screening tests and rule out potential contraindication in those who prepare for IFN-α therapy.

DILE is an autoimmune disease that shares similar clinical and serologic features with SLE (5). It is associated with the continuous use of certain drugs for months or years, and the symptoms may be relieved to some extent when the drugs are discontinued. Over 100 drugs have been identified to induce DILE, including procainamide, hydralazine, interferon, and tumor necrosis factor receptor antagonists (14). Although DILE and SLE share similar clinical characteristics, there are distinctions in their causative factors, management, and long-term prognosis (5, 15). In parallel with the prevalent use of biological agents, there has been a gradual increase in the incidence of DILE among patients with viral hepatitis and autoimmune disorders (5, 16). Some researchers suggested that drug intervention may be responsible for approximately 10% of SLE cases, and even a small dosage of medication can potentially contribute to the development and progression of DILE (16). However, the definite mechanism of the development of DILE remains elusive and may be related to numerous factors such as genetic predisposition, drug biotransformation, and epigenetic dysregulation of T cells (5). The incidence of IFN-α induced lupus had been historically estimated to be around 0.15–0.7% (17). It has been reported that patients receiving IFN-α for various indications, including chronic myelogenous leukemia, hepatitis C, hepatitis B, and malignant melanoma, are predisposed to DILE (2).The role of type I interferons (mainly IFN-α and IFN-β) in the pathogenesis and therpy of SLE have been elucidated in the latest review (18). Anifrolumab, a monoclonal antibody against type I interferon receptor, has been approved for the treatment of adult patients with active SLE and has shown excellent efficacy and safety profiles (19). To explore the features of IFN-α-triggered SLE in the past two decades, we conducted a comprehensive literature review. Our search was conducted on PubMed and CNKI, focusing on clinical case reports of IFN-α-induced lupus published between 1 January, 2003 and 1 February, 2023. The query terms were “lupus” and “IFN-α”. Only cases reported with full text available in English or Chinese in peer-reviewed journals were included in our analysis. In order to resolve any difference or uncertainty, two investigators were responsible for rechecking the source data respectively.

The clinical data extracted from the 13 case reports are summarized in **Table 2** (20–31). There were 5 males and 8 females, aged 16 to 57 years old. The primary diseases were chronic hepatitis C (CHC) in 8 cases, CHB in 4 cases, and aggressive central giant cell lesion in one case. At the onset of SLE, these patients had received IFN-α treatment for the duration of 2 to 36 months. 4 cases were treated with regular IFN-α, while 9 patients were treated with Peg-IFN-α. Based on the severity of SLE symptoms, patients were divided into two categories. The first group consisted of 5 cases with complicated lupus nephritis (LN), 7 cases with detectable serum anti-dsDNA antibodies, and 6 patients requiring high-dose prednisolone combined with immunosuppressive agents to induce remission. The second group exhibited a drug-induced lupus (DIL)-like syndrome. 7 cases presented with clinical manifestations implicating typical SLE symptoms, but did not involve important organs such as the central nervous system and kidneys. These patients were positive for ANA, but negative for SLE marker antibodies such as anti-dsDNA antibodies and Sm antibodies. They discontinued IFN-α and were treated with low-dose prednisolone and/or hydroxychloroquine (HCQ), and experienced rapid remission of SLE symptoms with no relapse. In addition, 2 other patients had achieved remission of DILE without glucocorticoid treatment.

**Table 2:** Summary of clinical characteristics of 13 prior case reports of SLE arising during IFN-α therapy from 2003 to 2023.

Reference No./Sex/age	Publication year	Indication for therapy	Symptoms and signs	Time to lupus	Laboratory data	IFN-α dose	Treatment	Outcome
[20]/M/50	2011	HCV	fevers, fatigue, arthralgias, facial rash	6 months	ANA, dsDNA, anti-SSA, leukopenia/lymphopenia	Peg IFN-α 180μg/wk	HCQ 0.2g Bid	Improvement
[21]/F/18	2009	HBV	photosensitivity, erythematous maculopapules, arthralgias	8 months	ANA 1/1000, anti-histone, hypocomplementemia	Peg-IFN α-2b 160μg/wk	HCQ 0.4g/d NSAIDS	Improvement
[22]/F/57	2010	HCV	fever, skin rash, pleuritis, parotid gland swelling	7 months	ANA + anti-SSA, anti-SSB, anti-Sm and dsDNA, proteinuria(2-4g/d), renal biopsy	Peg IFN-α-2b /wk	intravenous methylprednisone (1 g for 3 days) followed by oral dexamethasone (4.5 mg/day)	Improvement
[23]/F/48	2016	HCV	fever, arthralgias, oral ulcers, shortness of breath	2 months	ANA	Peg IFN-α	corticosteroids and hydroxychloroquine	Improvement
[24]/M/51	2014	HCV	fever, asthenia, joint pain, lymphopenia pain	49 weeks	ANA 1:80, anti-histone	Peg IFN-α 180μg/week	NSAID, corticosteroids, hydroxychloroquine	Improvement
[25]/F/53	2005	HCV	photosensitive malar rash, oral ulcers, arthralgias	10 months	ANA,anti-SSA, lymphopenia, hypocomplementemia	3MU 3x/wk	prednisone, hydroxychloroquine	Improvement
[26]/M/43	2008	HCV	maculopapular rash, oral ulcer, arthritis, bilateral pleural effusions, anemia	14 weeks	ANA 1/2560, anti-dsDNA, hypocomplementaemia, lymphopenia, renal biopsy	Peg IFN-α 180μg/week	prednisone, hydroxychloroquine, cyclophosphamide, IVIG, ICU therapy	Improvement
[27]/F/17	2016	HBV	fever, epilepsia, livedo reticularis, blurred visio in both eyes	12 months	ANA 1/320, Proteinuria (1.13g/24h), PLT 29, renal biopsy	2MU/qod	Prednisone, CTX, Depakine, mannitol	Improvement
[27]/F/26	2016	HBV	Rash, arthritis, weakness	36 months	ANA 1/1280, anti-histone	IFN-α	prednisone,hydroxychloroquine	Improvement
[28]/F/25	2012	HBV	Fever, arthritis	58 weeks	ANA 1/1000,dsDNA, proteinuria (2.17g/24h), renal biopsy	Peg IFN-α 180μg/week	prednisone, MMF	Improvement
[29]/F/16	2005	Giant cell lesion	malar rash, pleuritis	18 months	ANA 1/80 dsDNA, hypocomplementemia	3MU/m2/day	steroids	n.d
[30]/M/55	2006	HCV	arthritis, pleuro-pericardial effusion, ascites	44 weeks	ANA 1/640 dsDNA	Peg IFN α-2b 1.1 mcg/kg per week	Prednisone, diuretics, tacrolimus	Improvement
[31]/M/47	2009	HCV, HIV, HBV	truncal rash, abdominal pains and headache, proteinuria	27 months	ANA 1/1280 dsDNA, hypocomplementemia, renal biopsy	Peg IFN α	Prednisolone, MMF, haemodialysis	Improvement
Current case/M/31	Current	HBV	fever, headache	33 months	ANA 1/1000, dsDNA, WBC 2.73,PLT 49, hemolytic anemia, hypocomplementemia, hyperferritinemia,	Peg-IFN α-2a 180μg/wk	Prednisolone, CsA, CTX, etoposide	Improvement

ANA titer of antinuclear antibodies, dsDNA titer of anti-doublestranded DNA antibodies, anti- histone titer of anti- histone antibodies, SSA titer of anti-SSA antibodies, WBC (white blood cell) or or PLT (platelet) count given in thousands per mm^3^, renal biopsy renal biopsy performed and results compatible with lupus nephritis, MU million units;n.d, not determined.

In the present study, a case of a male patient developed IFN-α induced SLE complicated with HLH. His clinical and laboratory findings included fever, pancytopenia, hypocomplementemia, positive ANA and anti-dsDNA antibody, and an accumulated total score of 22, meeting the 2019 EULAR/ACR criteria for the diagnosis of SLE (9). The patient also exhibited fever, pancytopenia, hemophagocytosis in bone marrow, high levels of sCD25, and ferritin, hence fulfilling five out of the eight diagnostic criteria of HLH, as described in the HLH 2004 trial (7). Therefore, the patient was diagnosed with SLE that presented as HLH. The mechanism of HLH secondary to SLE is complex, as they share some similar features, but HLH can be characterized by hyperferritinemia, hypofibrinogenemia, and hypertriglyceridemia, in contrast with SLE. The in-hospital mortality was higher in SLE patients with HLH compared to those without HLH, thus it is crucial to make timely diagnosis and treatment for reducing mortality in HLH patients. Cytopenias are common manifestations of both HLH and SLE, and the presence of HLH may delay the diagnosis of underlying SLE. Therefore, it is important to perform immunologic testing for SLE in the setting of HLH and avoid diagnostic delays.

Although there were reported cases of IFN-α inducing SLE, no case of SLE complicated with HLH has been currently reported in CHB patients treated with IFN-α. The prevalence of SLE complicated with HLH has been reported to range from 0.9% to 4.6% without report of DILE (32). Given the challenge of differential diagnosis between SLE and HLH, and their overlapping manifestations, it can be difficult to determine the appropriate medication for these complicated conditions (33, 34). HLH is a rare immune disease and a potentially life-threatening disorder, characterized by cytokine storm and overwhelming inflammation and caused by abnormal activation and proliferation of lymphocytes, monocytes, and macrophages. It is manifested by fever, cytopenia, hepatosplenomegaly, hypertriglyceridemia, hyperferritinemia, and hemophagocytosis in the liver, spleen, bone marrow, or lymph nodes (6, 34). It can be caused by intrinsic genetic defects or extrinsic detrimental factors, which are denominated as primary or secondary hemophagocytic lymphohistocytosis (pHLH or sHLH), respectively (6). These external factors mainly consist of malignancies, infective viruses such as Epstein-Barr virus, cytomegalovirus, human immunodeficiency virus, or autoimmune disorders including SLE and adult-onset Still disease.

In the management of sHLH, it is vital to address the inflammation storm in accordance with the specific disease context (35). The administration of immunosuppressive agents and high-dose steroids is a promising approach for mitigating sHLH, but it must be cautious to prevent potential myelosuppression and secondary infections during inflammation storm abatement. The treatment of sHLH is intricate and may necessitate a multidisciplinary treatment (MDT) approach (36). The objective of HLH therapy is to manage the underlying condition and prevent further harm to the body (37). In this case, the patient achieved remission through a theapeutic regime consisting of high-dose methylprednisone, cyclosporine, and etoposide. Although HCQ is the cornerstone drug for the treatment of SLE, we did not use it because of the high myopia and partial visual field defects in the patient. Our experience was that closely monitor the patient’s blood routine, once the bone marrow suppression happens, immediately stop the etoposide therapy. Given the role of cytokine cascade in the pathogenesis of HLH, targeted inhibition of inflammatory factors has become a very promising treatment option in the era of biologic therapies. Anakinra,a recombinant humanised IL-1 receptor antagonist, is used in clinical practice to treat alternatively the patients with rheumatic sHLH and may allow for avoidance of etoposide (38, 39). Emapalumab, a human IFN γ blocking antibody, was efficacious in inducing remission of secondary HLH to sJIA or AOSD in patients (40, 41). However, until now both anakinra and emapalumab are not launched in mainland China therefore are not accessible currently. The effective treatment provides a tangible solution to IFN-α induced SLE complicated by HLH, and may represent an efficacious strategy for addressing above predicament.

In summary, we reported a rare case of a CHB patient suffering from IFN-α treatment induced SLE complicating HLH had achieved complete remission with a successful combination treatment of methylprednisone, cyclosporine, cyclophosphamide, and etoposide. Therefore, we have provided a novel and effective medication regimen for SLE complicating HLH, especially in the condition of IFN-α induced overwhelming inflammation in DILE.

## Data availability statement

The datasets presented in this study can be found in online repositories. The names of the repository/repositories and accession number(s) can be found in the article/supplementary material.

## Ethics statement

The study was conducted in accordance with the Declaration of Helsinki, and approved by the Ethics Committee of Tongji Hospital, Tongji Medical College, Huazhong University of Science and Technology. The patients/participants provided their written informed consent to participate in this study. Written informed consent was obtained from the individual(s) for the publication of any potentially identifiable images or data included in this article.

## Author contributions

ZZ, DN, and LY conceptualized and wrote the manuscript draft. WT, BJ, JL, KH critically revised the manuscript. All authors contributed to the article and approved the submitted version.
